# Genetics of eosinophilic esophagitis (EOE)

**DOI:** 10.1186/2045-7022-1-S1-S32

**Published:** 2011-08-12

**Authors:** Antonella Cianferoni

**Affiliations:** 1 The Children's Hospital of Philadelphia, University of Pennsylvania, Pediatrics Allergy and Immunology Division, Philadelphia, USA

## 

EoE is a global health condition, with an incidence of ~1 per 10,000 people in the US. Symptoms of EoE greatly impar quality of life. Individuals with EoE are predominantly young males with a high rate of atopic disease, and the diagnosis of EoE is made by endoscopy and biopsy findings of isolated eosinophils in the esophagus. Although the underlying cause remains unknown, EoE as other atopic diseases is recognized as complex genetic disorders, where multiple genes interact with each other and with the environment to trigger variable expression of the atopic phenotype. Accumulating evidence suggests that EoE has a strong familial association. Molecular analysis of esophageal biopsies and mouse models have indicated a clear role for the T helper 2 pathway, in particular interleukins 5 and 13, in this disease. In recent years, the genetics of atopy, asthma and EoE have been investigated using genome-wide linkage analyses and candidate gene association studies. Using such approach a single-nucleotide polymorphism (SNP) in the human eotaxin-3 gene has been found to be associated with EoE in about 15% of the patients. Such research approach in EoE as in atopy and asthma, while providing certain valuable insights into the genetics of atopic diseases, has achieved only limited success in identifying the genetic determinants of these related disorders. More recently, however, critical information provided by the Human Genome Project and the International HapMap Project has prompted the development of unprecedented genotyping technology and tools such as genome-wide association (GWA) studies to more comprehensively investigate the genetic basis of complex diseases such as atopy and EoE. In the past year, such research approach has led to the identification of single nucleotide polymorphisms in the gene encoding thymic stromal lymphopoietin (TSLP), and subsequently in the gene encoding its receptor, as disease susceptibility markers for EoE. Identification of this molecule and its receptor suggest the potential for new treatment options in the future.

